# The quality of antimalarials available in Yemen

**DOI:** 10.1186/1475-2875-4-28

**Published:** 2005-06-29

**Authors:** Ahmed Abdo-Rabbo, Amal Bassili, Hoda Atta

**Affiliations:** 1 Department of Pharmacology and Therapeutics, Faculty of Medicine and Health Sciences, University of Sana'a, Republic of Yemen; 2 Division of Communicable Diseases, Eastern Mediterranean Regional Office of the World Health Organization, Abdul Razzak Al Sanhouri Street, P.O.Box 7608, Nasr City, Cairo 11371, Egypt

## Abstract

**Background:**

Malaria has always been a major public health problem in Yemen. Several studies in developing countries have demonstrated ineffective and poor quality drugs including antimalarials. Therefore, quality assessment of antimalarial drugs is of crucial importance. This study aimed to assess the quality of antimalarials (chloroquine and sulfadoxine/pyrimethamine) available in Yemen and to determine whether the quality of these products was related to the level of the distribution chain at which the samples were collected or related to the manufacturers.

**Methods:**

Four samples from each antimalarial product were collected from each of the various levels of the distribution chain. One sample was kept with the research team. Two were tested at Sana'a and Aden Drug Quality Control Laboratories. The fourth was sent to the Centre for Quality Assurance of Medicines in Potchefstroom, South Africa, for analysis. Quality indicators measured were the content of the active ingredient and dissolution rate (for tablets only) in comparison to standard specifications for these products in the relevant pharmacopoeia.

**Results:**

The results identified several problems of sub-standard products within the drug distribution chain. They included high and low failures in ingredient content for chloroquine tablets and chloroquine syrup. There was some dissolution failure for chloroquine tablets, and high sulfadoxine/pyrimethamine tablets dissolution failures. Failures with the dissolution of the pyrimethamine were found at most of the collection points. No clear relationship neither between the quality products and the level of the distribution chain, nor between locally manufactured and imported products was observed.

**Conclusion:**

There are sub-standard antimalarial products circulating within the drug distribution chains in the country, which will have serious implications on the reduced therapeutic effectiveness and on the development of drug resistance. This appears to be due to non-compliance with Good Manufacturing Practice guidelines by manufacturers in the production of the antimalarials.

## Background

Malaria has always been a major public health problem in Yemen. About half of the population are at risk of malaria and there are several hundred deaths every year [1].

A preliminary, exploratory meeting of experts and other concerned parties was held in Geneva in October 1998. The meeting concluded, that poor quality antimalarials (AMs) posed a problem for malaria control, and therefore to public health, especially in countries where there is little or no drug regulatory infrastructure.

Treatment failure, associated with drug resistance may also be due to poor quality products, instability of products, or the use of counterfeit products. Several WHO-sponsored studies have demonstrated significantly the instability of products such as ergometrine, [2-4] and other essential medicines, during transport by sea, and also during road transport inland [5,6]. A WHO document on accelerated stability studies under simulated tropical conditions provides some indication of intrinsic stability of commonly used drugs [7]. A study in Sudan on stability of drugs in the tropics showed a considerable reduction in the concentration of the liquid dosage forms of certain preparations, whereas solid dosage forms were relatively stable [8]. A recent study in selected African countries identified several significant problems of sub-standard AM products within the drug distribution chain [9].

The problem of counterfeit drugs is well recognized in different parts of the developing world [10]. The use of ineffective and products of poor quality will not only endanger therapeutic treatment but also erode public confidence in national health programmes.

In most countries the quality of AMs is rarely independently evaluated and the local capacity for independent drug-quality assurance is worst where the disease burden is the highest. AMs of poor quality might contribute to the emergence of resistance. It is therefore, important to consider product quality when dealing with the problem of antimalarial resistance.

In Yemen, drug registration is overseen by the Supreme Board of Drugs and Medical Appliances (SBD&MA). There are two national reference laboratories for drug quality control, one in Sana'a and the other in Aden.

The Drug Fund (DF) tender for public sector drugs centrally. Suppliers deliver drugs to the four public regional stores in the country. Lahej Governorate obtain their medical supplies from Aden regional medical stores (RMS) through Lahej central medical stores (CMS). The existing district medical stores receive supplies from Lahej CMS and distribute them to the health facilities within their district. Sometimes drugs may be supplied directly from Lahej CMS to the health facilities.

In view of the need to clarify the nature and magnitude of the problem of AM quality in the country, it was decided to carry out a study in a selected Governorate to evaluate the extent of the problem and guide designing suitable intervention strategies.

This study aimed at assessing the quality of the commonly used AMs in Yemen, particularly, chloroquine and sulfadoxine/pyrimethamine and to determine whether the sub-standard quality of these products was related to the level of the distribution chain at which the samples were collected or to the manufacturer. It also aimed at evaluating the performance of the national reference laboratories, by comparing their results to those obtained from an international reference laboratory in South Africa.

## Methods

Chloroquine phosphate 250 mg tablets (CQT), chloroquine base 50 mg/5 ml syrup (CQS), and sulfadoxine/pyrimethamine 500 mg/25 mg tablets (SPT) were sampled and evaluated. They were chosen because they are the most widely used AMs in Yemen and included in Yemen Treatment Guidelines and Essential Drugs List [11].

The study adopted the same methods used by the WHO to assess the quality of antimalarials in malaria endemic countries [9]. Therefore, samples were collected at various levels of the drug distribution chain in the public and private sector in Lahej Governorate, which is one of the highest malaria-endemic areas in Yemen. Samples were collected from 3 medical stores, the Governorate hospital, 4 district hospitals/health centres, 9 health units and 5 private sector pharmacies. The total samples collected from all levels of the drug distribution were 15 CQS, 25 CQT, and 10 SPT, for details see Table 1.

**Table 1:** Summary sample collection grid for quality study

**Facilities**	**#**	**CQT**	**CQS**	**SPT**	**Total**
RMS	1	1	1	1	3
GMS	1	1	1	1	3
DMS	1	2	-	-	2
GH	1	1	1	1	3
RH/HC	4	6 (1 exp)	3 (2 exp)	2	11
HU	9	9	4 (1 exp)	-	13
PH	5	5	5	5	15
Total	22	25	15	10	50

RMS = Regional Medical Store, Aden;

GMS = Central Medical Stores, Lahej Governorate DMS = District Medical Stores; GH = Governorate General Hospital; RH/HC = Rural Hospital/Health Centre; HU = Health Unit; PP = Private Pharmacy/Drug Store; exp = Expired CQT = Chloroquine tablets; CQS = Chloroquine syrup; SPT= Sulfadoxine Pyrimethamine tablets.

The sampling was performed as follows. Four districts, in addition to the capital of the governorate, were selected by simple random sampling. Samples from the Governorate hospital and the hospitals of the four districts were studied (there is only one hospital or health centre in each district). Nine health units which are working and easy to reach were chosen (most health units are difficult to reach because of long distance or bad roads). Samples were also collected from only one district medical stores because there are no medical stores in the other districts as the hospital/health centre store functioning as a hospital store, pharmacy and also supply the district health units.

Samples were also collected from 5 private pharmacies in the governorate.

One sample was collected from the governorate capital and one sample from each of the studied districts. The pharmacies were selected by systematic random sampling.

Chloroquine syrup in both public and private sector was available in bottles of 100 or 120 ml. Chloroquine tablets were available in strips of 10 tablets or tins/bottles of 1000 tablets. Sulfadoxine/pyrimethamine tablets were present in strips of 3 or 10 tablets each.

Four bottles of chloroquine syrup, four samples of chloroquine tablets (20 tablets/sample) and four samples of sulfadoxine/pyrimethamine tablets (20 tablets/sample) were taken by convenience sampling. Each preparation was from the same batch.

Four samples from each batch were collected from each private community pharmacy/drug store and each level of the public drug distribution chain. These samples were divided as follow:

One was kept with the research team; one was tested at Sana'a Drug Quality Control Laboratory (Sana'a DQCL); one was tested at Aden Drug Quality Control Laboratory (Aden DQCL); one was sent to Eastern Mediterranean Regional office of the World Health Organization (WHO/EMRO) and then forwarded to the Centre for Quality Assurance of Medicine (CENQAM) in Potchefstroom, in South Africa for analysis.

The following information was recorded during field survey for each drug sample on a serially numbered data collection form: location of sample collection point (name of the facility); facility type; name of drug (brand or generic); strength and dosage form; date of manufacture; date of expiry; description of packaging material and any remarks on storage; date of sample collection; name of person in charge of institution and name of person collecting the sample

The collected samples of tablets were transferred from their original containers into amber glass bottles with screw cap seals. Syrups were left in their original containers. The containers were carefully labeled at the point of collection with sticker containing the same information on the original container, particularly, drug name, batch number, facility name, sampling date and sample form number. Each sticker was given a unique sampling identification code.

All testing was carried out at Sana'a and Aden DQCLs. For verification the same samples were tested in CENQAM Laboratories. Samples were analysed according to pharmacopoeial specifications to assess the quality of the products. The quality indicators measured were the content of the active ingredient for syrup, and dissolution and content for tablets.

There is no analytical monograph in the United States Pharmacopoeia (USP) or British Pharmacopoeia (BP) for the assay of CQS. CENQAM used a high pressure liquid chromatography (HPLC) method which was developed and validated in-house [9]. Sana'a and Aden DQCLs used the method of PHARCO Pharmaceuticals-an Egyptian pharmaceutical manufacturer. In this method the absorbance of both standard and test solutions was measured by UV-spectroscopy at 329 nm.

The content of CQ in tablets was determined by UV-spectrophotometry in accordance with an adapted method for CQT of the USP 2003:424. The method consists of recording the UV absorbance of the sample at a wavelength of 343 nm and comparing it to the absorbance of a reference standard in a concentration range representing a 100% label claim at the same wavelength.

The content of SP was determined by means of the HPLC method for the assay of SPT described in the USP 2003:1734. The mobile phase was adjusted to satisfy the system suitability criteria of the USP for chromatographic systems.

The general method and apparatus of the USP were used for testing the dissolution characteristics of both CQT and SPT according to their respective dissolution monographs. The dissolution parameters for CQT in the USP 2003:424 and for SPT in the USP 2003:1734 were followed.

## Results

Table 2 shows the laboratory results of CQS tested in the three reference laboratories. As CQS has no official monograph in the USP or BP, an acceptance criteria of 90–110 of the label claim was adopted. Only one product did not comply with the adapted criteria and tested too high i.e. had some "high" content failure. This high percentage ingredient failure (116.2%) was recorded at Tor-Albeh rural hospital/health centre and manufactured by YEDCO, Republic of Yemen.

**Table 2:** The laboratory results of chloroquine syrup tested at the three reference laboratories

**CHLOROQUINE SYRUP (Limit: 90–110%)**						
**Code**	**CENQAM**		**DQCL-Sana'a**		**DQCL-Aden**	
	
	***Assay (%)***	***% RSD***	***Assay (%)***	***% RSD***	***Assay (%)***	***% RSD***
***CQS/RMS***	104.1	3.20	96.20	0.60	102.0	0.36
***CQS/SMS***	105.7	1.10	100.5	1.01	100.5	3.71
***CQS/LGH***	109.0	3.20	99.4	1.94	101.2	1.77
***CQS/MRH-1***	91.1	3.50	101.2	2.65	102.7	3.80
***CQS/HRH***	100.0	0.85	95.3	0.65	107.7	0.71
***CQS/TRH-EXP***	116.2	3.00	98.5	1.22	99.4	0.75
***CQS/HU-1***	108.3	4.90	102.2	0.78	102.7	4.11
***CQS/HU-7***	107.8	4.60	98.5	0.3	97.6	6.33
***CQS/HU-8***	107.4	4.30	102.6	2.01	100.8	1.30
***CQS/HU-9***	109.9	1.20	98.1	0.83	95.9	9.15
***CQS/PP-1***	92.6	2.70	97.2	3.82	94.8	4.56
***CQS/PP-2***	90.7	2.50	102.6	2.10	96.4	2.10
***CQS/PP-3***	107.0	4.90	97.5	3.10	100.7	2.8
***CQS/PP-4***	98.0	4.30	101.3	2.10	99.7	1.45
***CQS/PP-5***	100.3	4.60	101.6	1.14	102.4	1.36

CENQAM = Centre for Quality Assurance of Medicine, South Africa

RSD = Relative standard deviation

DQCL = Drug Quality Control Laboratory

RMS = Regional Medical Store, Aden; GMS = Central Medical Stores, Lahej Governorate; DMS = District Medical Stores; GH = Governorate General Hospital; RH/HC = Rural Hospital/Health Centre; HU = Health Unit; PP = Private Pharmacy/Drug Store; exp = Expired; CQS = Chloroquine syrup.

The acceptance criteria for CQT (phosphate) in the USP are 93–107% of the stated amount per unit. Most failures were "low" failures i.e. sample contents below the minimum recommended levels for the products The lowest percentage failures in ingredient content were recorded for 3 products at Al-Raga health unit in Tor-Albaheh district, central medical stores in Lahej and regional medical stores in Aden. The three products were manufactured by YEDCO, Republic of Yemen. The highest percentage ingredient contents failure were found at Al-Mosimir rural hospital and Akan health unit in Al-Mosimir district. They were manufactured by YEDCO, Republic of Yemen, and PHARMED, the Netherlands respectively.

Dissolution testing was done on 6 units and % relative standard deviation (% RSD) of < 5 % was observed for most of the products tested. The exceptions were CQT/HU-7 (5.3%) and CQT/HU-9 (7.6%).

The dissolution acceptance criteria for CQT of the USP were applied. CQT have a Q-value (the quantity of dissolved active specified in the monograph, expressed as a percentage of the labeled content) of 75% in 45 minutes and, according to the acceptance table of USP 2003:2161, the dissolution of each of the six units tested should not be less than 80% (Q+5). The product found at Al-Meghafah health unit, Toben district (CQT/HU-7) and the product found at Al-Fiosh health unit, Toben district (CQT/HU-9) did not comply with the criteria. These two products are manufactured by ALKALOIDA Chemical Co. Ltd., Hungary (See Additional file 1, showing the results of chloroquine tablets testing in the three reference laboratories).

The content criteria for SPT in the USP 2003:1734 are 90–110 of the label claims for both actives. The percentage of SP contents in tablets for all studied SPT samples complied with the criteria with respect to both actives.

Dissolution testing was done on 6 units and a high % RSD was observed for pyrimethamine in some of the products tested. In general the % RSD for the pyrimethamine was higher than for the sulfadoxine content in most of the products.

The dissolution acceptance criteria for SPT of the USP were applied. SPT have a Q-value of 60% in 30 minutes for both actives and according to the acceptance table of USP 2003:2161, the dissolution of each 6 units tested should not be less than 65% (Q+5) for both the actives.

However, all products complied with the criteria with regard to sulfadoxine, only two products complied with the criteria for both actives.

The first product was found at Eskander private drug store in Toben district (SPT/PP-1), which manufactured by Intas Pharmaceuticals Ltd., India. The other product was found at Al-Ikhlas private drug store in Al-Mosimir district (SPT/PP-2), and manufactured by Roche, Switzerland.

The other products did not comply with the criteria for pyrimethamine. Also only 1 unit of product SPT/CMS [67.1% – 3.3 (% RSD) = 63.8%] did not comply with the criteria for pyrimethamine(ç).

There was no significant difference between the national DQC and CENQAM reference laboratories regarding the active ingredients for CQS, CQT, and SPT, or CQT dissolution rate. On the other hand, there was a significant difference between them regarding the SP dissolution whereby only 30% of SP tested were fulfilling the USP criteria in CENQAM laboratory compared to all tested products in the national DQCLs (Table 3).

**Table 3:** Comparison between national reference laboratories and CENQAM Reference laboratory regarding the quality screening of AM drugs

	**CENQAM *% fulfilling the USP content criteria***	**Sana'a DQCL *% fulfilling the USP content criteria***	***P1*-*value***	**Aden DQCL *% fulfilling the USP content criteria***	***P2*-*value***	***P3*-*value***
**CQS Assay**	93.3 (14/15)	100 (15/15)	1	100 (15/15)	1	*1*
**CQT Assay**	80 (20/25)	92 (23/25)	0.41	84 (21/25)	1	*0.66*
**CQT dissolution**	92 (23/25)	100 (25/25)	0.47	96 (24/25)	1	*1*
**SP Assay**	100 (10/10)	100 (10/10)	1	100 (10/10)	0	*1*
**SPT dissolution**	20 (2/10)	100 (10/10)	0.005*	100 (10/10)	0.005*	*1*

***P1***: p-value of Chi-Square test for comparing the proportion of tested products fulfilling the USP content criteria in CENQAM versus Sana'a reference laboratory.

***P2***: p-value of Chi-Square test for comparing the proportion of tested products fulfilling the USP content criteria in CENQAM versus Aden reference laboratory.

***P3***: p-value of Chi-Square test for comparing the proportion of tested products fulfilling the USP content criteria in Sana'a versus Aden reference laboratory.

Table 4 shows a comparison between the quality of antimalarial drugs obtained from this study and that of 7 malaria endemic countries.

**Table 4:** Comparison between country results: percentage failure of samples

	**Yemen**	**Sudan**	**Gabon**	**Ghana**	**Mali**	**Kenya**	**Mozambique**	**Zimbabwe**
**CQS Assay**	**6.7**	26.6	0	5.0	66.7	25.0	25.0	13.3
**CQT assay**	**20**	5.2	29.0	66.7	47.3	42.8	20.0	57.1
**CQT dissolution**	**8**	12.5	5.8	20.0	5.2	28.6	6.7	7.1
**SP assay**	**0**	0	0	37.5	0	0	5.5	10.0
**SP dissolution**	**70**	80	-	75.0	100	91.7	100	100

## Discussion

The quality control performed in this study was utilized to ensure that the batch of tested AMs complies with its specification and is fit for its intended in terms of efficacy, safety and acceptability.

CQS had content failure rate of average 6.7%. The failure was "high" failure. Content failure for CQT was very significant and averaged at 20%. Failures were "low" and "high" failures. SPT content complied with criteria with respect to both actives in the 3 reference laboratories.

Dissolution failure rates for CQT were 8%. SPT had problems mostly with the dissolution of the pyrimethamine component of the formulation, and averaged at 80% for the SPT samples analysed.

The data presented in this report indicates there is a problem of sub-standard AMs available at the public health facilities and circulating in the market. The main problem seems to be samples below the lower limit of specification. The most significant results were the low content failures for CQT content and SPT dissolution. The failures detected in this study indicate a serious problem that warrants further investigation and intervention.

Quality problems were not limited to a particular distribution level but recorded at different levels. Also there were failures among locally made products as well as foreign products. Further investigation of this phenomenon will be important since it is easier for national drug regulatory authorities to act and correct problems that involve domestic manufacturers.

The discrepancy between the national reference laboratories and CENQAM results in ingredient content failure for CQ and SPT dissolution, can only be taken as indicative, but not conclusive and should be carefully investigated to identify the causes and rectify them. As the three laboratories use the same methodology for quality screening of drugs, the possible reasons for such discrepancy could be personnel errors and/or inadequate calibration of the instruments used.

In comparison with other malaria endemic countries that adopted the same methodology in evaluating the quality of antimalarial drugs [9], the following comments could be drawn from the study:

• CQS content failure in Yemen is relatively low and is almost comparable to Ghana's. On the other hand, it is significantly lower than other countries such as: Sudan, Mali, Kenya, Mozambique and Zimbabwe.

• CQT content failure in Yemen is higher than Sudan, comparable to Gabon, and Mozambique, but significantly lower than Ghana, Mali, Kenya and Zimbabwe.

• CQT dissolution failure in Yemen is relatively low. It is lower than Sudan, Ghana, Kenya, but comparable to Gabon, Mali and Mozambique.

• SP content showed no failure, as in most other malaria endemic countries. Similarly, SP dissolution failure was as high as other malaria endemic countries.

## Conclusion

Problems of sub-standard AMs exist in different studied districts, within the drug distribution chain in Lahej, Yemen and produced by both domestic and foreign manufacturers.

Percentage failure of samples based on ingredient content is 6.7% for CQS and 20% for CQT and in dissolution failure 8% for CQT and 70% for SPT, cannot be ignored. This will have serious implications not only on the reduced therapeutic effectiveness of AMs but also on the development of drug resistance.

In view of potential danger that sub-standard AMs could already be posing in the fight against malaria, an intervention plan should be developed immediately. This could involve setting up quality surveillance systems within drug regulatory authorities in the country, supporting manufacturers to improve Good Manufacturing Practice (GMP) compliance and promote effective managing drug supply.

The lessons learnt from this pilot study could be valuable in any future investigations, and interventions to improve the quality of AMs and other medicines in use in domestic and international markets.

## Recommendations

• Promoting good procurement practices in the public sector by registration or pre-qualification of suppliers, i.e. purchasing from reliable and approved sources.

• More emphasis should be given to testing for initial quality, the routine testing of new supplies, ensuring adherence of manufacturers and suppliers to GMP.

• Antimalarial drugs should be stored and distributed in appropriate conditions in harbours, stores and health facilities as well as at the level of the end users.

• Measures should be taken to stop the illegal importing or smuggling of drugs.

• Ensure that people in charge of drug management supply and quality control are competent and receive training, a measure which would be cost-effective.

• Although the cost of antimalarial drugs is important, good quality antimalarial drugs are more important than cheaper poor drugs.

## Authors' contributions

**Dr Ahmed Abdo-Rabbo: **protocol development, field work implementation, data collection and analysis and interpretation of data; drafted the article.

**Dr Amal Bassili **assisted in protocol development, supervision on the progress of the project, assistance in data analysis and interpretation; revision of the manuscript.

**Dr Hoda Atta **assisted in protocol development, supervision on the progress of the project; revision of the manuscript. All authors have given final approval to the version to be submitted for publication.

## Supplementary Material

Additional File 1 Results of chloroquine tablets testing in the three reference laboratories Click here for file

Additional File 1 Results of chloroquine tablets testing in the three reference laboratories Click here for file
