# Supplementary material for: The quality of antimalarials available in Yemen
Source: Malar J. 2005 Jun 29;4:28. doi: 10.1186/1475-2875-4-28 (PMC1192817; doi:10.1186/1475-2875-4-28)
Supplement: Additional File 1 — Results of chloroquine tablets testing in the three reference laboratories [file 1475-2875-4-28-S1.doc]

**Additional file 1: The laboratory results of chloroquine tablets tested in the three reference laboratories**

| **CHLOROQUINE TABLETS (Limit: Content 93-107%, Dissolution > 80%)** | | | | | | | | | | | | |
| --- | --- | --- | --- | --- | --- | --- | --- | --- | --- | --- | --- | --- |
| **Code** | **CENQAM** | | | | **DQCL-Sana’a** | | | | **DQCL-Aden** | | | |
|  | **Assay**  **%** | **%**  **RSD** | **Diss.**  **%** | **%**  **RSD** | **Assay**  **%** | **%**  **RSD** | **Diss.**  **%** | **%**  **RSD** | **Assay**  **%** | **%**  **RSD** | **Diss.**  **%** | **%**  **RSD** |
| **CQT/RMS** | **97.6** | **0.34** | **96.9** | **4.10** | **95.0** | **4.13** | **99.5** | **5.60** | **91.9** | **2.25** | **92.9** | **1.21** |
| **CQT/CMS** | **94.4** | **0.88** | **92.7** | **2.50** | **92.8** | **1.10** | **92.2** | **4.90** | **90.2** | **0.84** | **94.8** | **0.57** |
| **CQT/HMS-1** | **98.7** | **1.00** | **99.3** | **1.90** | **94.6** | **3.70** | **90.5** | **3.70** | **95.7** | **0.08** | **106.3** | **1.77** |
| **CQT/HMS-2** | **95.9** | **1.60** | **88.8** | **1.70** | **101.3** | **6.44** | **92.9** | **3.91** | **96.8** | **0.25** | **93.7** | **2.86** |
| **CQT/LGH** | **96.1** | **1.30** | **97.5** | **1.70** | **99.0** | **2.50** | **97.0** | **3.60** | **99.2** | **0.24** | **102.7** | **1.73** |
| **CQT/MRH-1** | **101.1** | **3.30** | **98.3** | **1.10** | **98.0** | **6.60** | **107.2** | **1.81** | **110.3** | **0.02** | **102.3** | **1.75** |
| **CQT/MRH-2** | **97.9** | **1.20** | **92.7** | **1.50** | **98.5** | **0.38** | **99.9** | **1.30** | **96.1** | **0.00** | **100.2** | **4.70** |
| **CQT/RRH** | **94.4** | **1.70** | **94.3** | **1.50** | **96.9** | **066** | **98.8** | **0.82** | **95.4** | **1.35** | **87.5** | **6.52** |
| **CQT/HRH** | **101.7** | **1.40** | **102.5** | **1.40** | **100.4** | **0.70** | **95.4** | **0.91** | **99.4** | **0.16** | **96.5** | **2.20** |
| **CQT/TRH-1** | **95.9** | **0.30** | **98.9** | **0.90** | **97.8** | **1.72** | **95.2** | **2.25** | **94.1** | **0.22** | **87.7** | **1.80** |
| **CQT/TRH-2** | **96.2** | **0.41** | **96.1** | **2.20** | **101.9** | **0.51** | **103.5** | **1.00** | **97.4** | **0.13** | **93.3** | **1.25** |
| **CQT/HU-1** | **100.2** | **1.30** | **91.7** | **1.70** | **103.0** | **0.90** | **104.6** | **0.97** | **100.5** | **0.77** | **100.4** | **1.52** |
| **CQT/HU-2** | **99.8** | **0.92** | **99.6** | **3.00** | **107.7** | **0.19** | **102.5** | **0.77** | **95.8** | **0.54** | **90.8** | **3.10** |
| **CQT/HU-3** | **99.3** | **0.39** | **96.7** | **2.20** | **97.9** | **0.50** | **103.4** | **3.70** | **93.6** | **1.28** | **105.2** | **2.16** |
| **CQT/HU-4** | **99.2** | **0.39** | **97.8** | **1.70** | **100.1** | **0.80** | **103.3** | **1.90** | **96.5** | **0.85** | **99.9** | **0.74** |
| **CQT/HU-5** | **90.3** | **1.10** | **93.8** | **3.20** | **95.4** | **4.70** | **94.4** | **2.30** | **89.5** | **0.72** | **97.6** | **1.63** |
| **CQT/HU-6** | **94.0** | **0.89** | **97.6** | **0.92** | **101.0** | **0.80** | **105.2** | **1.40** | **96.2** | **2.65** | **100.7** | **2.33** |
| **CQT/HU-7** | **98.2** | **0.89** | **84.9** | **5.30** | **103.1** | **0.14** | **99.5** | **1.71** | **102.6** | **0.13** | **103.6** | **1.20** |
| **CQT/HU-8** | **96.1** | **0.88** | **99.3** | **1.50** | **99.6** | **1.63** | **99.4** | **0.40** | **95.0** | **1.70** | **87.5** | **1.25** |
| **CQT/HU-9** | **98.0** | **1.80** | **74.5** | **7.60** | **100.3** | **1.92** | **88.0** | **4.90** | **101.5** | **0.21** | **73.1** | **2.90** |
| **CQT/PP-1** | **101.0** | **3.20** | **95.6** | **0.30** | **99.8** | **0.62** | **95.8** | **0.51** | **93.2** | **0.16** | **113.3** | **0.07** |
| **CQT/PP-2** | **99.5** | **0.20** | **99.9** | **0.53** | **99.4** | **1.60** | **103.5** | **4.30** | **101.8** | **1.78** | **102.1** | **3.75** |
| **CQT/PP-3** | **98.0** | **1.90** | **99.2** | **2.30** | **101.1** | **1.98** | **97.7** | **5.20** | **96.9** | **0.08** | **98.6** | **1.38** |
| **CQT/PP-4** | **99.9** | **0.69** | **100.3** | **1.80** | **100.9** | **1.25** | **98.4** | **2.22** | **103.7** | **0.32** | **105.6** | **0.65** |
| **CQT/PP-5** | **95.6** | **1.00** | **93.6** | **1.90** | **99.5** | **0.40** | **96.2** | **2.50** | **93.6** | **1.43** | **96.8** | **0.77** |

RMS = Regional Medical Store, Aden; GMS = Central Medical Stores, Lahej Governorate; DMS = District Medical Stores; GH = Governorate General Hospital; RH/HC = Rural Hospital/Health Centre; HU = Health Unit; PP = Private Pharmacy/Drug Store; exp = Expired;  CQT=Chloroquine tablets.
