# Supplementary material for: The quality of antimalarials available in Yemen
Source: Malar J. 2005 Jun 29;4:28. doi: 10.1186/1475-2875-4-28 (PMC1192817; doi:10.1186/1475-2875-4-28)
Supplement: Additional File 1 — Results of chloroquine tablets testing in the three reference laboratories [file 1475-2875-4-28-S2.doc]

**Additional file 2: The laboratory results of sulfadoxine/pyrimethamine tested in three reference laboratories**

| **SULFADOXINE/PYRIMETHAMINE TABLETS**  **(Limit: Content 90-110%, Dissolution < 60% in 30 min)** | | | | | | | | | | | |
| --- | --- | --- | --- | --- | --- | --- | --- | --- | --- | --- | --- |
| **Code** | **Active** | **CENQAM** | | | | **DQCL-Sana’a** | | | | **DQCL-Aden** | |
|  |  | **Assay**  **%** | **%**  **RSD** | **Diss.**  **%** | **%**  **RSD** | **Assay**  **%** | **%**  **RSD** | **Diss.**  **%** | **%**  **RSD** | **Assay**  **%** | **Diss.**  **%** |
| **SPT/RMS** | **Sulfa** | **97.6** | **0.44** | **96.5** | **1.10** | **95.0** | **3.63** | **97.0** | **0.56** | **98.0** | **100.6** |
|  | **Pyrimeth.** | **102.4** | **0.41** | **54.6*** | **0.96** | **91.0** | **0.90** | **78.0** | **0.27** | **99.0** | **99.0** |
| **SPT/CMS** | **Sulfa** | **101.8** | **0.25** | **98.8** | **2.60** | **102.8** | **0.15** | **100.1** | **0.70** | **103.3** | **101.4** |
|  | **Pyrimeth.** | **101.3** | **0.95** | **67.1** | **3.30** | **97.2** | **0.97** | **87.7** | **0.72** | **100.4** | **105.0** |
| **SPT/LGH** | **Sulfa** | **99.7** | **0.75** | **99.7** | **2.20** | **102.0** | **0.50** | **99.0** | **0.80** | **100.6** | **104.5** |
|  | **Pyrimeth.** | **101.3** | **0.83** | **23.5*** | **23.00** | **91.9** | **1.22** | **84.6** | **0.72** | **100.4** | **101.1** |
| **SPT/RRH** | **Sulfa** | **99.7** | **0.18** | **97.0** | **2.10** | **102.8** | **1.14** | **105.1** | **1.30** | **98.9** | **102.3** |
|  | **Pyrimeth.** | **100.5** | **0.49** | **25.2*** | **5.20** | **92.3** | **1.25** | **86.2** | **0.75** | **96.6** | **97.5** |
| SPT/TRH-1 | **Sulfa** | **100.6** | **0.93** | **93.0** | **2.80** | **93.0** | **7.10** | **91.4** | **0.12** | **100.3** | **98.8** |
|  | **Pyrimeth.** | **103.1** | **0.96** | **25.3*** | **6.40** | **96.3** | **3.96** | **88.0** | **0.70** | **100.6** | **99.9** |
| **SPT/PP-1** | **Sulfa** | **98.8** | **0.45** | **99.1** | **1.60** | **101.2** | **0.64** | **95.8** | **0.40** | **102.4** | **98.8** |
|  | **Pyrimeth.** | **98.0** | **0.36** | **73.0** | **1.60** | **95.8** | **1.50** | **87.8** | **0.88** | **101.3** | **100.7** |
| **SPT/PP-2** | **Sulfa** | **100.5** | **1.20** | **97.9** | **0.58** | **102.5** | **0.67** | **101.7** | **1.55** | **100.6** | **97.7** |
|  | **Pyrimeth.** | **100.6** | **0.88** | **98.1** | **1.70** | **95.4** | **0.64** | **106.7** | **2.60** | **98.7** | **104.6** |
| **SPT/PP-3** | **Sulfa** | **99.9** | **0.66** | **93.8** | **2.80** | **102.9** | **0.37** | **103.2** | **0.22** | **100.7** | **95.7** |
|  | **Pyrimeth.** | **104.5** | **0.36** | **54.7*** | **2.20** | **94.5** | **0.76** | **87.7** | **0.51** | **99.6** | **100.4** |
| **SPT/PP-4** | **Sulfa** | **101.5** | **0.27** | **98.0** | **1.20** | **99.6** | **4.40** | **94.8** | **0.15** | **99.8** | **102.7** |
|  | **Pyrimeth.** | **101.3** | **0.14** | **22.9*** | **6.10** | **97.3** | **1.52** | **79.5** | **0.70** | **99.9** | **102.4** |
| **SPT/PP-5** | **Sulfa** | **99.3** | **1.90** | **94.5** | **1.80** | **100.6** | **1.15** | **85.7** | **0.98** | **101.2** | **98.5** |
|  | **Pyrimeth.** | **101.3** | **1.70** | **46.5*** | **3.40** | **94.0** | **0.70** | **95.5** | **0.09** | **99.0** | **98.6** |

 RMS = Regional Medical Store, Aden; GMS = Central Medical Stores, Lahej Governorate; DMS = District Medical Stores; GH = Governorate General Hospital; RH/HC = Rural Hospital/Health Centre; HU = Health Unit; PP = Private Pharmacy/Drug Store; exp = Expired; SPT= Sulfadoxine-pyrimethamine tablets.
